# Beam commissioning of the first clinical biology‐guided radiotherapy system

**DOI:** 10.1002/acm2.13607

**Published:** 2022-04-28

**Authors:** Bin Han, Dante Capaldi, Nataliya Kovalchuk, Eric Simiele, John White, Daniel Zaks, Lei Xing, Murat Surucu

**Affiliations:** ^1^ Department of Radiation Oncology Stanford University Stanford California USA; ^2^ RefleXion Medical Hayward California USA

**Keywords:** BGRT, commissioning, RefleXion

## Abstract

This study reports the beam commissioning results for the first clinical RefleXion Linac.

**Methods**: The X1 produces a 6 MV photon beam and the maximum clinical field size is 40 × 2 cm^2^ at source‐to‐axis distance of 85 cm. Treatment fields are collimated by a binary multileaf collimator (MLC) system with 64 leaves with width of 0.625 cm and y‐jaw pairs to provide either a 1 or 2 cm opening. The mechanical alignment of the radiation source, the y‐jaw, and MLC were checked with film and ion chambers. The beam parameters were characterized using a diode detector in a compact water tank. In‐air lateral profiles and in‐water percentage depth dose (PDD) were measured for beam modeling of the treatment planning system (TPS). The lateral profiles, PDDs, and output factors were acquired for field sizes from 1.25 × 1 to 40 × 2 cm^2^ field to verify the beam modeling. The rotational output variation and synchronicity were tested to check the gantry angle, couch motion, and gantry rotation.

**Results**: The source misalignments were 0.049 mm in *y*‐direction, 0.66% out‐of‐focus in *x*‐direction. The divergence of the beam axis was 0.36 mm with a y‐jaw twist of 0.03°. Clinical off‐axis treatment fields shared a common center in *y*‐direction were within 0.03 mm. The MLC misalignment and twist were 0.57 mm and 0.15°. For all measured fields ranging from the size from 1.25 × 1 to 40 × 2 cm^2^, the mean difference between measured and TPS modeled PDD at 10 cm depth was −0.3%. The mean transverse profile difference in the field core was −0.3% ± 1.1%. The full‐width half maximum (FWHM) modeling was within 0.5 mm. The measured output factors agreed with TPS within 0.8%.

**Conclusions**: This study summarizes our specific experience commissioning the first novel RefleXion linac, which may assist future users of this technology when implementing it into their own clinics.

## 1 INTRODUCTION

This study describes the experiences of our institution in commissioning the RefleXion X1 machine (RefleXion Medical Inc., Hayward, CA, USA), a novel linac designed for image‐guided radiation therapy (IGRT), stereotactic body radiation therapy (SBRT), and eventually biology‐guided radiotherapy (BgRT). The X1 machine consists of an enclosed O‐ring gantry linear accelerator (linac), a positron emission tomography (PET) system, a kilo‐voltage computed tomography (kVCT) system, and a mega‐voltage (MV) imaging system. The X1 machine has significant differences compared with conventional C‐arm linacs but shares some components with the Tomotherapy machine.^1^ Therefore, we adopted the American Association of Physicists in Medicine (AAPM) Task Group 148^2^ methodologies in the commissioning process. The goal of this work is to summarize the acceptance testing and beam commissioning of the X1 machine and share our experience in beam data acquisition and the TPS modeling validation, in order to establish reference data for future commissioning efforts.

**FIGURE 1:**
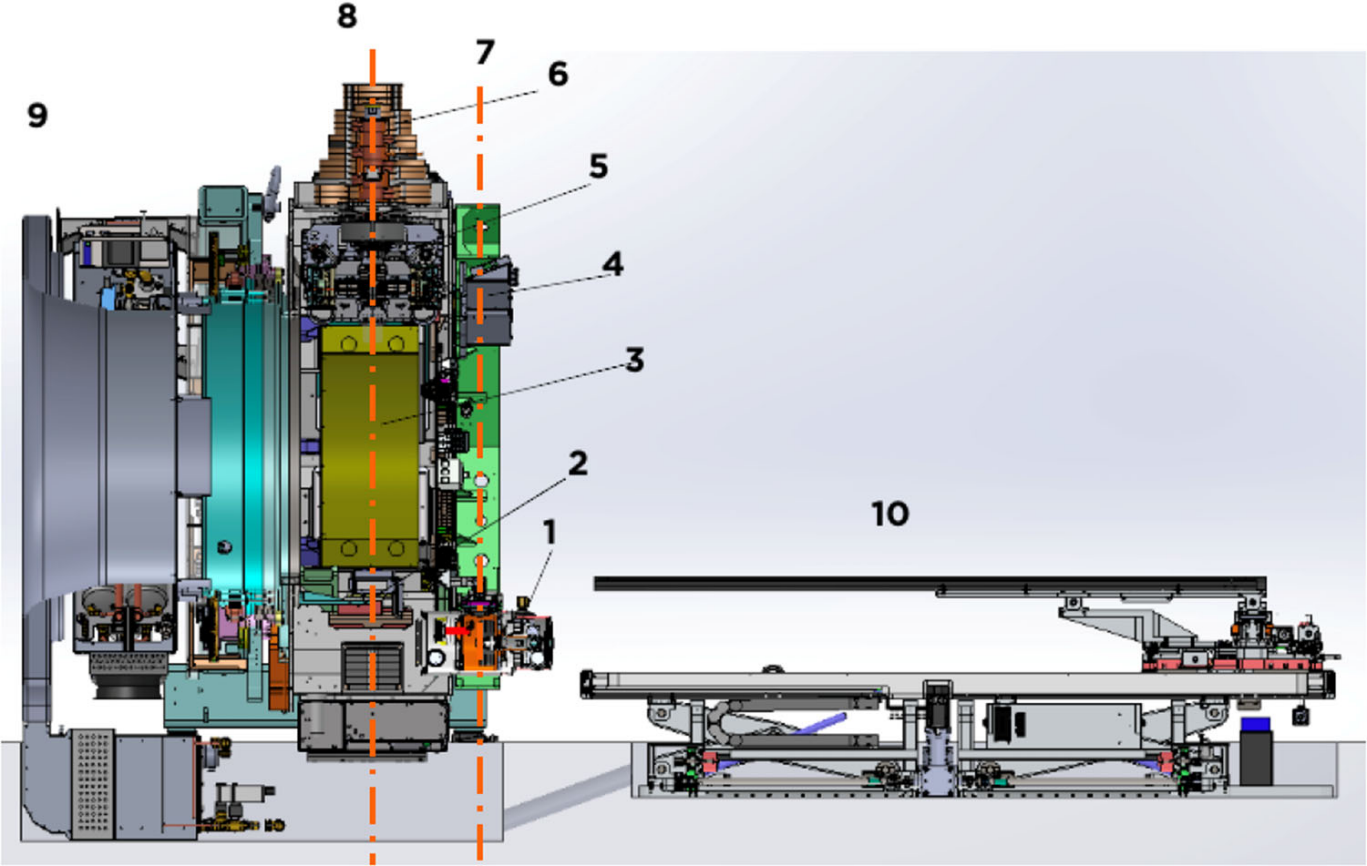
Section view of the RefleXion X1 linac with components: (1) kVCT X‐ray tube; (2) MV EPID; (3) PET detectors; (4) kVCT detector; (5) primary collimators; (6) 6MV linac; (7) kVCT plane; (8) MV and PET plane; (9) cooling system; and (10) couch

The main features of this new treatment device designed for BgRT are summarized in Table 1. Major components of the system are shown in Figure 1. The PET system detects real‐time positron emission signals that represent tumor motion and guides the machine to deliver high‐dose radiation through a binary multileaf collimation (MLC) system. The linac produces a 6 MV flattening filter‐free (FFF) photon beam and its collimation system consists of a binary MLC with 64 leaves and two pairs of y‐jaws located above and below the MLC. The MLC leaf side focuses on the source and each leaf provides a 0.625 cm opening in the *x*‐direction at the source‐to‐axis distance (SAD) of 85 cm, giving a total of 40 cm opening when all MLC leaves are retracted. The y‐jaw pairs move simultaneously to open 1 or 2 cm in the *y*‐direction at SAD of 85 cm. The nominal dose rate is 850 MU/min. The kVCT for image guidance is a 16‐slice fan‐beam CT. The PET detectors are dual 90° arcs at the same axial plane to the MV beam. The gantry rotation speed is constant at 60 rotations per minute (RPM) for kVCT scans, MV treatment delivery, and PET signal acquisition.

**TABLE 1:** Features of the RefleXion X1 treatment device

**RefleXion X1 features**	**Description**
Ring‐mounted gantry rotation	60 RPM
Source to isocenter distance	85 cm
Treatment beam	6 MV FFF
Nominal dose rate	850 MU/min
MLC design	Binary, 64, 6.25 mm leaf width
Maximum clinical field size	40 × 2 cm
Couch motion during delivery	Step (2.1 mm) and shoot
kVCT	Fan beam CT, 16‐slice
PET detectors	Dual 90° arcs

In a typical workflow on the RefleXion X1 IGRT treatment, the patient is first positioned using external wall lasers outside of the gantry housing. After laser setup confirmation, the couch then translates into the bore by a specific distance, moving the patient to the kVCT imaging plane, which is 614 mm superior to the laser center. kVCT images are then acquired to perform 3D‐to‐3D match to the treatment planning CT. A 5D couch shift is performed to accurately position the patient (roll is adjusted by offsetting the gantry angle), and the couch is then translated 386 mm superior into the bore to the MV and PET plane for treatment delivery. The treatment delivery is in a step‐and‐shoot fashion with the couch translating 2.1 mm each step in one or four passes for intensity‐modulated radiotherapy (IMRT) or SBRT delivery, respectively.

This study describes the commissioning and calibration tests that are exhaustive and largely based on relevant AAPM^2^ and IAEA^3^ documents. Because the physical design, especially the small field feature of RefleXion X1 differs from Tomotherapy and other C‐arm linacs, we have shared our specific experience in the setup and use of QA devices for this specific unit.

## 2 MATERIALS AND METHOD

The mechanical and dosimetric tests were performed according to the AAPM Protocol Task Group 148.^2^ The commissioning of the kVCT, PET, and MV imaging systems is out of the scope of this study. More detailed TPS commissioning following the AAPM TG‐53,^4^ and TG‐244^4^ are summarized in a different study.

### 2.1 Mechanical alignments

#### 2.1.1 Source alignments

We have performed the center alignment check of the radiation source in the *y*‐direction against the y‐jaw to ensure the misalignment within 0.3 mm. The procedure used a 2 mm y‐jaw opening that was moved in 11 steps along the *y*‐direction. A narrow y‐jaw setting amplified the sensitivity of this test. The beam was turned on for a fixed amount of time with the y‐jaw opening shifted from −14 to +14 mm off‐axis in a 2 mm step. At each step, the output was measured with an Exradin A17 chamber (Standard Imaging, Middleton, WI, USA) with a stationary long active volume and a linear response over a length sufficient to measure the output of the shifted beams. The A17 chamber was setup directly on the treatment couch with the long axis aligned with the machine's IEC‐*y*‐axis and the center of the chamber at the treatment isocenter location. The output was plotted as a function of axial jaw shift. The source was aligned with the y‐jaw when beam output was at its peak, as determined by a parabolic fit to the data.

The centering of the source in the *x*‐direction was checked against the MLC position using the MLC tongue and groove (T&G) effect following the test in V.B.1.b in TG‐148.^2^ This effect is caused by the T&G design of the leaves that prevents a direct path for radiation to pass through when adjacent leaves are closed. A consequence of this design is a difference in the fluence output if two adjacent leaves open in sequence versus a simultaneous opening. The vendor specifies a maximum out‐of‐focus tolerance of 2%. The T&G effect is minimized if the MLC is focused on the source, therefore it can be used to test the source to MLC alignment. Crossline water tank beam scan data from a diode detector were used to collect output profiles with all even‐numbered MLC leaves opened and then with all odd‐numbered MLC leaves opened. This delivery sequence maximized the T&G effect. To test the *x*‐alignment of the source, the odd‐numbered leaf profiles and even‐numbered leaf profiles were added and then divided by an open‐field profile that was collected with all MLC leaves open. The symmetry of the normalized T&G profile was assessed to test the centering accuracy of the source to the MLC in the *x*‐direction.

#### 2.1.2 MLC and y‐jaw alignments

The alignment of the y‐jaw with the beam plane was checked to assure that the central transverse axis of the treatment beam intersects the rotational axis perpendicularly. This test ensures that when the gantry is at 0° and the beam diverges symmetrically around the plane of the gantry rotation, the y‐jaws are parallel to the plane of rotation. The divergence of the beam axis from perpendicular at isocenter should be within 0.5 mm, and the physical jaw twist should be less than 0.5°. A film was positioned horizontally between solid water plates (at the depth of 2 cm) and 21 cm below the isocenter. A half open beam with all MLC on the left side opened and y‐jaw set to 2 cm was delivered with gantry positioned at 0°. The same half‐open beam was then delivered to the same film with the gantry rotated 180°. The film result was assessed to detect the beam center and beam edge to calculate the jaw divergence and twist based the calculations, which are in the sections V.B.1.c and V.B.1.d in TG148.^2^

The centering accuracy in *y*‐direction of multiple off‐axis clinical treatment fields was tested. A film was placed perpendicularly to the beam axis at an 85 cm source‐to‐film distance under a stationary vertical field. Three 1.25 × 2 cm and four 1.25 × 1 cm beams with 425 MU were delivered with the gantry at 0°. Profiles taken across the different treatment slice widths at off‐axis positions in the film were used to determine the field centers, and the variations in *y*‐direction were calculated.

The lateral alignment and twist of the MLC relative to the center of rotation were tested. The y‐jaw should also be parallel to the plane of rotation. The AAPM TG148 suggested tolerances are less than 1.5 mm for the MLC offset at the isocenter and the twist less than 0.5°. A film‐based test was used to test these two parameters. A film was positioned at isocenter and two central MLC leaves opened in addition to two off‐center leaves and the y‐jaws were set at 2 cm. The film was exposed with the gantry at 0°. The gantry was then moved to 180° and the beam was delivered with only the two off‐center leaves opened. The film result was analyzed to detect the MLC offset and twist.

#### 2.1.3 Couch alignment and positional accuracy verification

The couch level was checked using a digital level at six different positions, three longitudinal and three transverse to verify if the difference was within the tolerance of 0.2°. The couch motion and digital readout accuracy were checked relative to the sagittal and lateral lasers to verify that (a) the couch has less than 1 mm lateral divergence from the sagittal laser over 700 mm inferior–superior couch displacement; (b) the couch has less than 2 mm lateral divergence from the laser over the vertical range of motion allowed by the bore limits; and (c) without weight the couch sag over 700 mm, inferior–superior couch displacement is less than 5 mm. We have performed the couch lateral alignment center test to verify that the position of the couch was centered around the radiation isocenter. The distances from the sagittal laser to the two lateral most distal points on the couch were measured to ensure they were within 1 mm difference.

#### 2.1.4 Laser alignment tests

**FIGURE 2:**
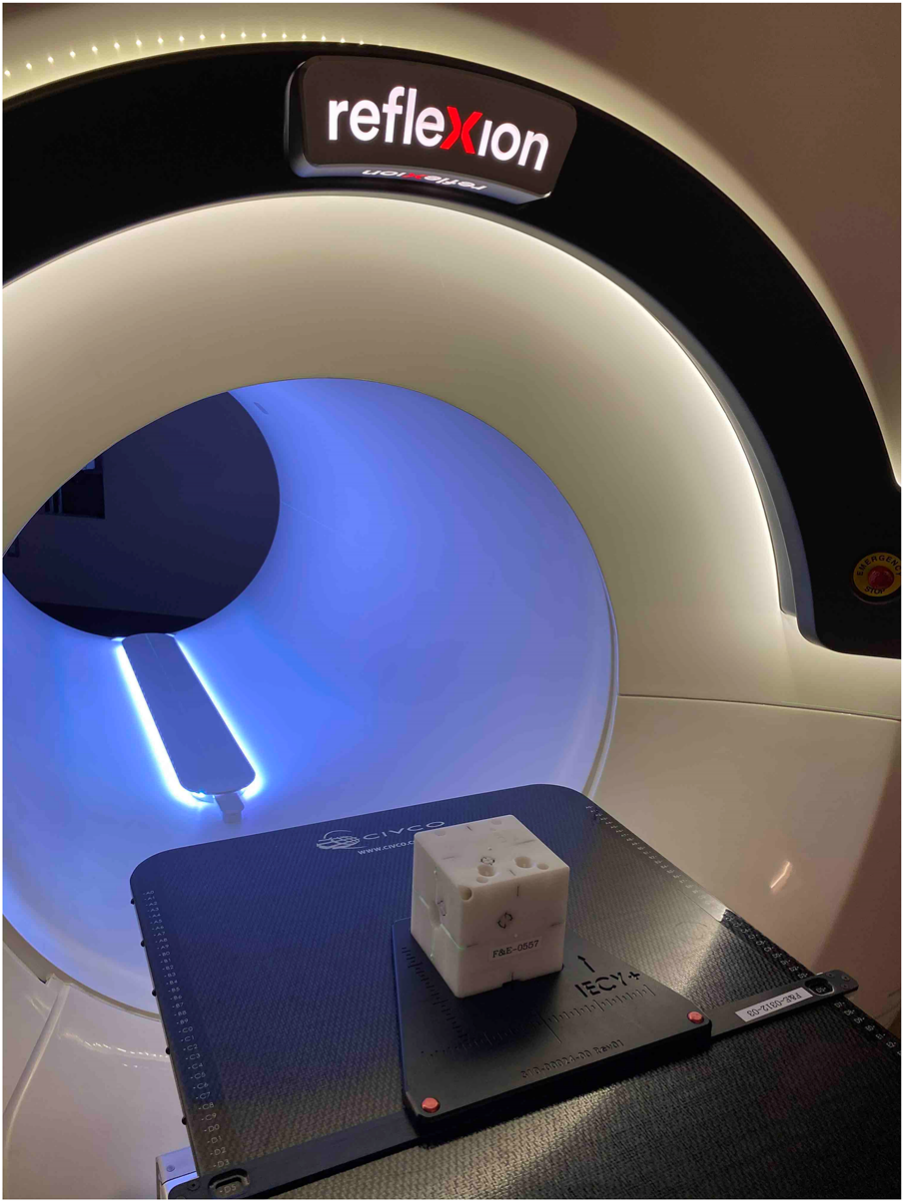
Imaging ball cube phantom setup at the external laser center

The RefleXion X1 machine offers no ODI or light field alignments like a regular C‐arm machine. Therefore, the initial patient alignment is purely dependent on the external wall laser. A total of four cross‐lasers were installed, including a ceiling laser, two lateral wall lasers, and a superior sagittal laser through the gantry bore. A mechanical test was performed to evaluate the divergence of all lasers at the external setup laser center and for the superior sagittal laser over a distance of 100 cm from the setup laser center to the MV field center. As shown in Figure 2, utilizing an imaging phantom that was set to travel 100 cm from the lateral wall lasers to the radiation isocenter, MV images were taken at gantry 0° and 90° to obtain the phantom offset from laser to radiation. The purpose of this test was to ensure that all lasers were accurately aligned 100 cm superior to the radiation isocenter.

#### 2.1.5 Other mechanical tests

The binary MLC positions accuracy and repeatability tests were performed by taking multiple MV images with the MLC field set to open only odd leaves and only even leaves. Starshots with gantry rotation were also performed to test the coincidence of radiation beams to a common isocenter under gantry rotation. A piece of film was sandwiched in between two 30 × 30 × 5 cm^3^ solid water blocks. The solid water block was set up on its side (30 × 30 cm in the axial plane) and the film was aligned to the lateral and ceiling lasers. The solid water block was moved 100 cm superior into the bore to the MV beam plane. 200 MU was delivered with the beam size to 1.25 × 2 cm^2^ at gantry angles of 0°, 72°, 144°, 216°, and 288°. The film was analyzed using RIT software (Radiological Imaging Technology, Colorado Springs, CO, USA) to find the minimum tangent circle radius, which quantifies the coincidence of the isocenter with the gantry.

### 2.2 Beam characteristic and dosimetry tests

#### 2.2.1 Percentage depth dose and relative beam profiles

Percentage depth dose (PDD) and profile scans were performed using the Edge diode detector (Sun Nuclear, Melbourne, FL, USA) in a Blue Phantom Helix water tank (IBA dosimetry GmbH, Germany). The Edge diode has an active detection area of 0.8 × 0.8 mm^2^. A total of 22 scans were collected as the input scans for generating the dose computational model of the treatment planning system (TPS). The clinical beams are using two different y‐jaw widths 1 and 2 cm only. Twenty in air profile scans and two PDD water scans of 40 × 2 and 40 × 1 cm^2^ open fields were performed as the TPS modeling input. Additional in water PDD and profile scans were acquired to verify the TPS accuracy and capture the beam characteristic and baseline for future QA. Table 2 summarizes the TPS modeling scans and verification scans. After the TPS modeling, the dose was calculated for all corresponding stationary plans to water phantom and compared with measurement. PDD at 10 cm depth (PDD_10_), normally served as an indicator of the beam quality, was also compared and the impact of beam collimation on PDD_10_ was evaluated.

**TABLE 2:** TPS modeling and verification scans

**Medium**	**MLC**	**Jaw**	**Depth (cm)**	**Scan type**	**No. of scans**	**TPS modeling**
Air	Single leave	2 cm	SAD	Transverse profile	8	Input
Air	double leave	2 cm	SAD	Transverse profile	8	Input
Air	40 cm	1, 2 cm	SAD	Transverse/axial profiles	4	Input
Water	40 cm	1, 2 cm		PDD	2	Input
Water	40, 20, 10, 5, 2.5, 1.25 cm	1, 2 cm		PDD	12	Verification
Water	40, 20, 10, 5, 2.5, 1.25 cm	1, 2 cm	1.5, 5, 10, 15, 20 cm	Transverse/axial profiles	120	Verification

#### 2.2.2 Relative output factors

As most of the treatment fields for RefleXion machine are in the category of small fields because of lack of lateral equilibrium in the *y*‐direction. The choice of detector could lead to dosimetric uncertainties for small‐field output factor measurements.^5^, ^6^, ^7^, ^8^, ^9^ The relative output factors were measured in plastic water at a depth of 10 cm using the Edge diode and a tissue equivalent W2 scintillator detector (Standard Imaging, Middleton, WI, USA).^10^, ^11^ The Edge diode detector was used for fields in *x*‐direction (MLC) from 1.25 to 40 cm. The W2 1 × 1 scintillator in a cylindrical shape with a length of 1 mm and a diameter of 1 mm was used for smaller fields in *x*‐direction (MLC) from 0.625 to 10 cm. For the diode and W2 measurement, the exact placement of the detector center at the radiation field was achieved by delivering radiation multiple times with small couch movements (0.2 mm step size) in the transverse and longitudinal directions to find the maximum detector reading, which corresponds to the exact centering of the detector in the beam. The output factor was then calculated as the ratio of detector reading acquired at the beam center under a field of interest to the reading acquired under the reference field, which is 10 × 2 cm^2^. TPS modeled output factors were also calculated to a water phantom for comparison.

#### 2.2.3 Rotational output constancy and synchronicity

The nominal dose rate for the RefleXion X1 is 850 MU/min and it is important to monitor the dose output fluctuation with the gantry rotation. The output of stationary deliveries at gantry angles of 0°, 90°, 180°, and 270° were measured using a Tomodose (Sun Nuclear, Melbourne, FL, USA) diode array mounted to the gantry. The dose output and profile constancy were checked. An A14SL ion chamber (Standard Imaging, Middleton, WI, USA) placed at the isocenter in a cylindrical phantom was used to monitor the rotational output variation. A 40 × 2 cm^2^ beam was delivered with the gantry rotating continuously at 60 RPM for more than 30 min. The measured current during the delivery was recorded to analyze the rotational output constancy with suggested tolerance limit of 2% of the mean output.

A synchronicity plan was designed to evaluate the accurate transmission of beams through the MLC to the isocenter in clinical step‐and‐shoot mode with gantry rotating at 60 RPM, and couch advancing 2.1 mm each step. The plan delivers 2 × 2.5 cm^2^ beam at five gantry angles and three couch positions. With film attached to the surface of a cylindrical phantom, the result is 15 rectangular exposures with equal 14.5° angular separation and 42 mm longitudinal distance. The tolerances for offset and angular deviation are 0.5 mm and 0.5°.

#### 2.2.4 IMRT verification

The RefleXion system is delivering the IMRT with the gantry rotating at 60 rpm, the MLC leaves transiting at 100 times per second, and the couch moving 2.1 mm per step. To further evaluate the complex integrated plan delivery accuracy, we measured the AAPM TG119^2^ head and neck (HN) plan and prostate plan using the ArcCHECK diode array system (Sun Nuclear Corp. Melbourne, FL, USA). The measurement results were compared with the TPS calculations using gamma analysis (3%, 2 mm).

## 3 RESULTS

### 3.1 Mechanical checks

#### 3.1.1 Source alignments

**FIGURE 3:**
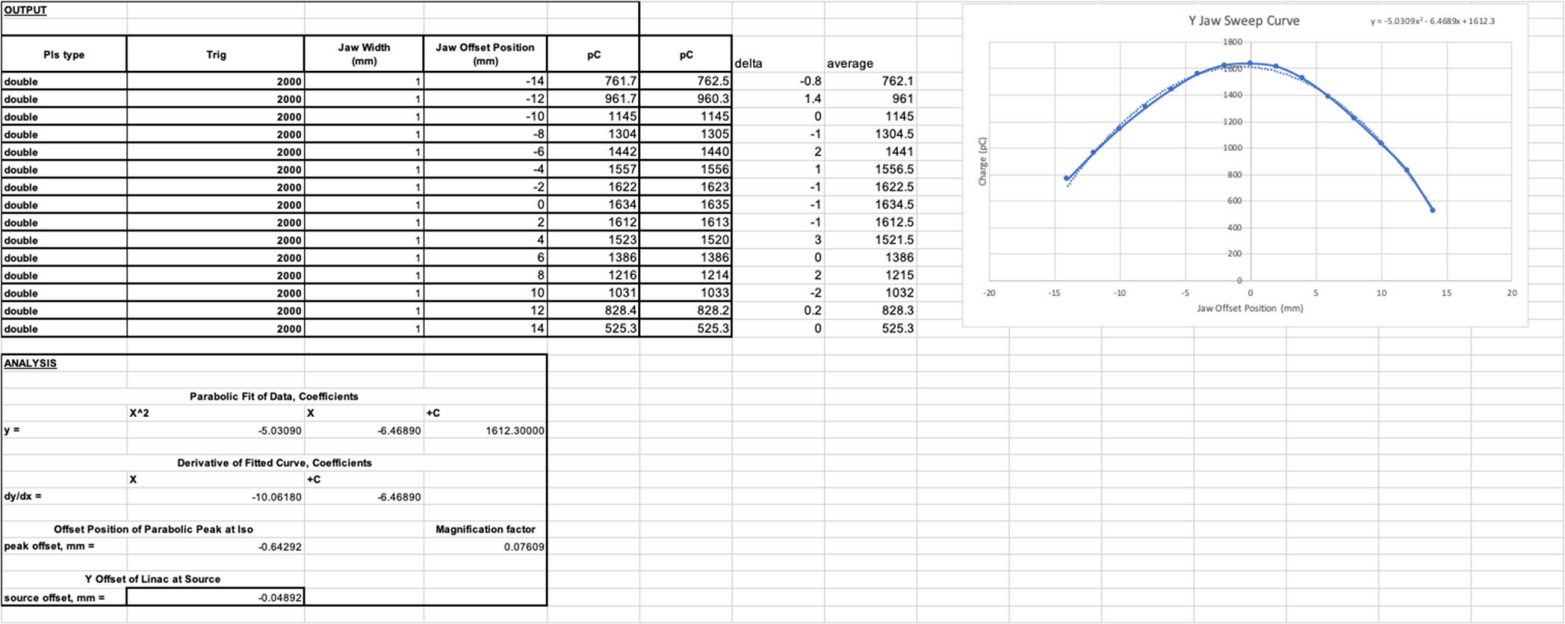
Y‐jaw versus output sweep curve (the solid line) and the corresponding parabolic fit curve (the dashed line). The actual source misalignment is 0.049 mm

The source to y‐jaw alignment test was performed using an Exradin A17 chamber to measure the output of the sweeping beams. After one round of alignment tuning, the measured output is plotted as a function of axial jaw shift in Figure 3 as the solid line. The source is aligned with the y‐jaw when beam output is at its peak, as determined by a parabolic fit (dashed line) to the data. The peak offset was −0.64 mm at the isocenter calculated from the measured y‐jaw sweep curve. The y‐jaw focus point is located 7 cm above the x‐ray source (i.e., 92 cm above the isocenter), which means that the source shift is magnified by a factor of 92/7 at the isocenter. Therefore, projected back to the source location, the actual source misalignment was 0.049 mm within the AAPM TG‐148 suggested 0.3 mm tolerance.

**FIGURE 4:**
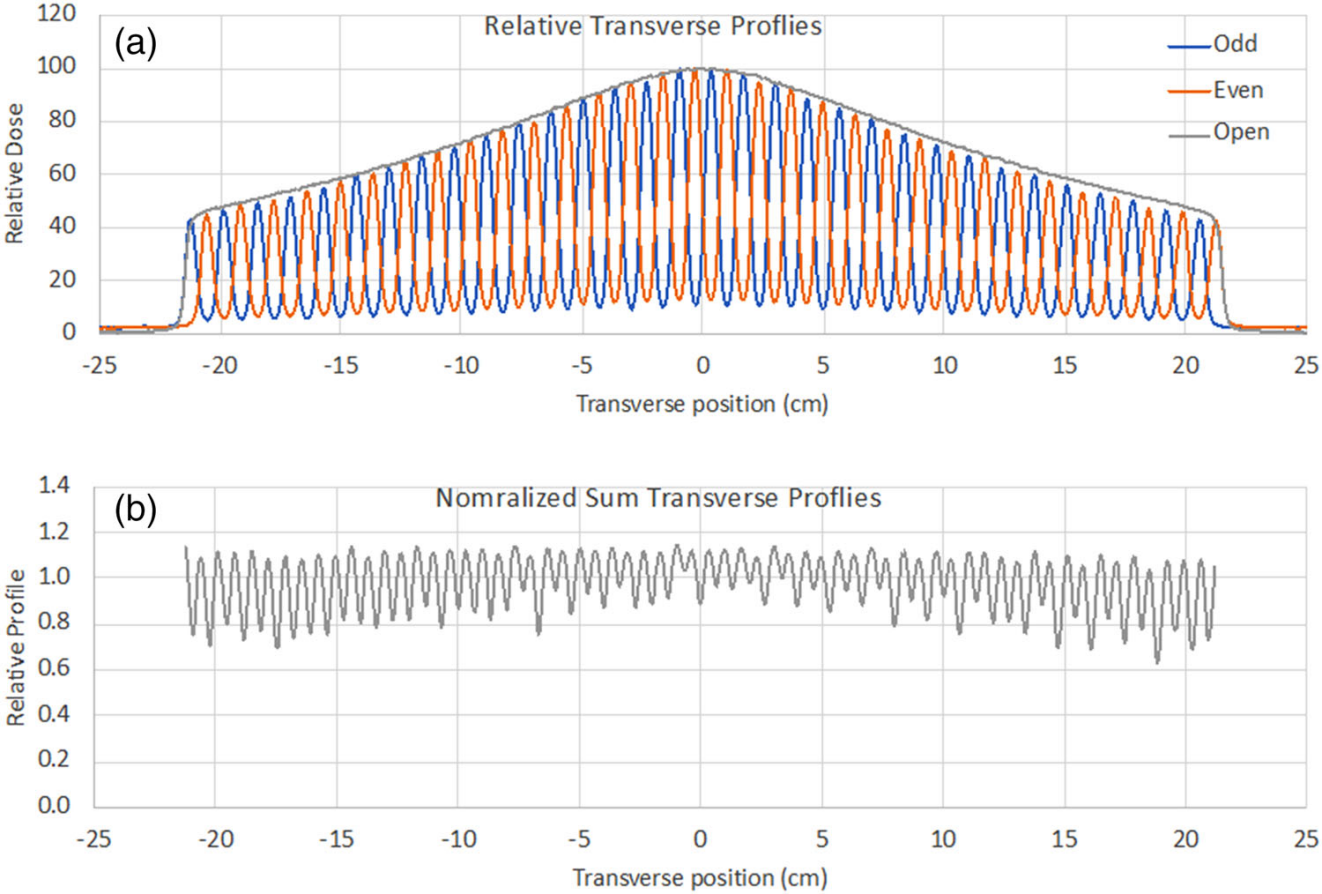
MLC T&G and open field profiles (upper). Normalized sum profile for even and odd profiles (lower). The out‐of‐focus value calculated from the image was 0.66%

The position of the source in the *x*‐direction was checked against the MLC position using an MLC tongue and groove (T&G) test. Transverse profiles at 1.5 cm depth with even‐numbered MLC leaves opened, odd‐numbered MLC leaves opened, and all MLC opened were measured in the water tank with a diode detector. The odd‐numbered and even‐numbered leaf profiles were added and then normalized by the open‐field profile to calculate the out‐of‐focus of the source to the MLC. All T&G profiles are shown in Figure 4 and the out‐of‐focus value was 0.66% within AAPM TG‐148 suggested tolerance of 2%. The out‐of‐focus was calculated using the formula (1) in the AAPM TG‐148 report.^2^

#### 3.1.2 MLC and y‐jaw alignments

**FIGURE 5:**
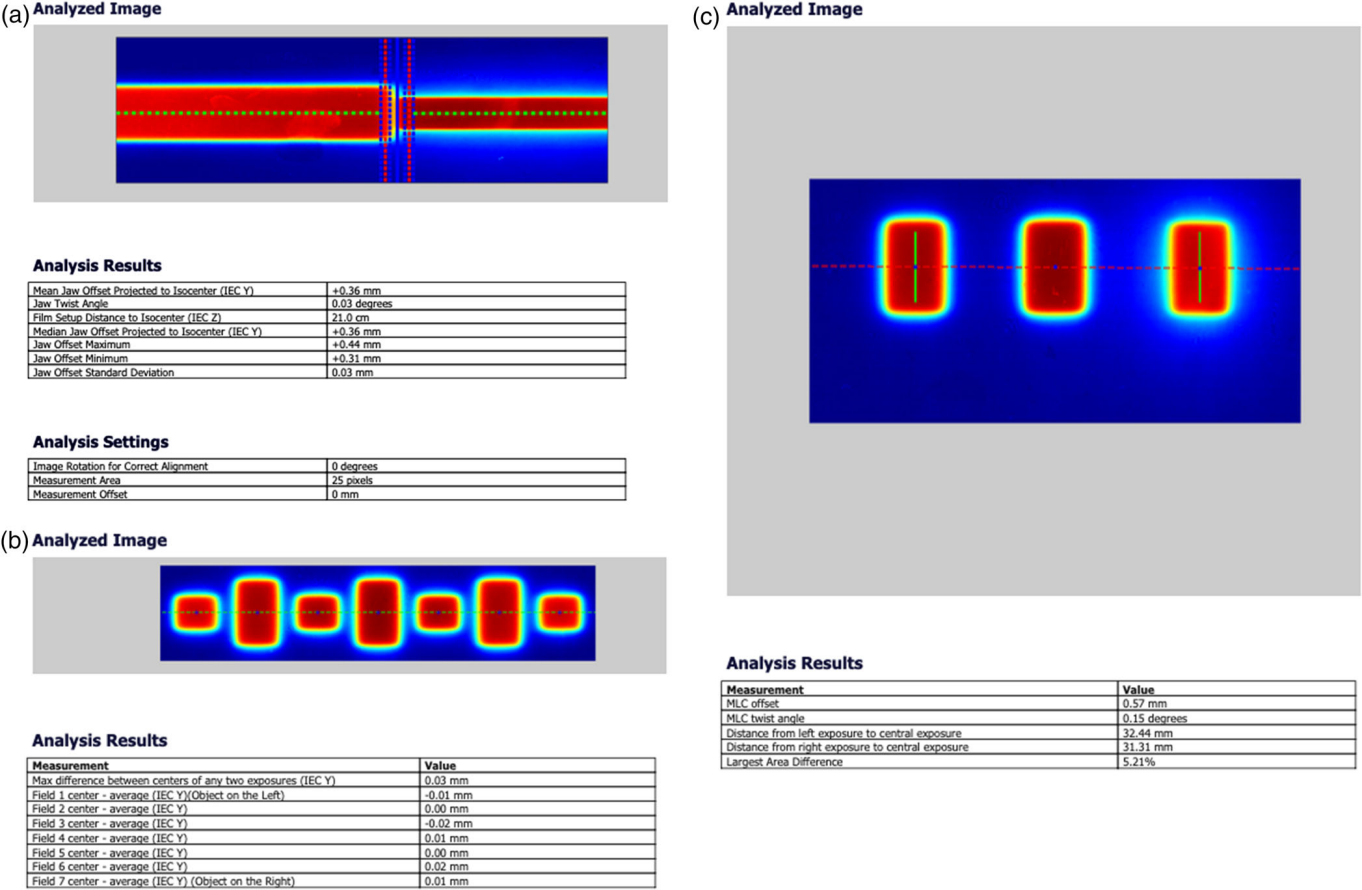
Film exposure testing for (a) the y‐jaw alignment relative to the plane of beam rotation, (b) treatment field centering, and (c) MLC lateral alignment test

Figure 5A shows the developed film result of the y‐jaw divergence and twist relative to the beam rotational axis. The divergence of the beam axis from perpendicular at isocenter was 0.36 mm, which was within the 0.5 mm tolerance. The y‐jaw twist result was 0.03° within the 0.5° tolerance, which showed that the y‐jaw was parallel to the plane of rotation.

Another film exposure test was performed to verify all clinical off‐axis treatment fields share a common center in *y*‐direction. Figure 5B shows the developed film results of three 1.25 × 2 cm^2^ and four 1.25 × 1 cm^2^ beams. The field center variations in *y*‐direction were within 0.03 mm at isocenter, which was within the suggested tolerance of 0.5 mm.

The lateral alignment of the MLC relative to the center of rotation was tested by exposing a film positioned at the isocenter. The film was first exposed with the gantry at 0° and two central MLC leaves (31 and 32) and two off‐center leaves (26 and 27) opened, and then exposed with gantry 180° and only the two off‐center leaves (26 and 27) opened. Figure 5C shows the MLC offset was 0.57 mm and twist was 0.15°, which are within the tolerance of 1.5 mm and 0.5°, respectively.

#### 3.1.3 Couch alignment and positional accuracy verification

The couch leveling accuracy was checked using a digital level placed at six different positions, three longitudinal and three transverse. Without applying any rotation to the couch, readings at all positions were within 0.2° accuracy. The couch was moved longitudinally over 700 mm and the lateral displacement of the couch was 0.8 mm relative to the sagittal and ceiling lasers. The couch was moved vertically over the range allowed by the bore limits and the lateral divergence relative to the sagittal and ceiling lasers was 0.9 mm. The couch sag without weight was 1.1 mm across 700 mm inferior–superior couch movement. The couch sag with 70 kg of weight was 1.9 mm across 700 mm inferior–superior couch movement. The accuracy of the centering position of the couch was 0.4 mm when measuring the distance from the sagittal laser to the two lateral most distal points on the couch.

#### 3.1.4 Laser alignment tests

Utilizing an image phantom that was set to travel 100 cm from the green lasers to the radiation isocenter, MV images were taken at gantry 0° and 90° to obtain the phantom offset from laser to radiation. The phantom offset from laser to radiation center measured in the MV image are 0.8, −0.2, and −0.2 mm in IEC *x*, *y*, and *z* directions, respectively, within the suggested 1 mm tolerance. The result indicated that wall laser is accurately aligned to the radiation isocenter with known 100 cm distance.

#### 3.1.5 Other mechanical tests

**FIGURE 6:**
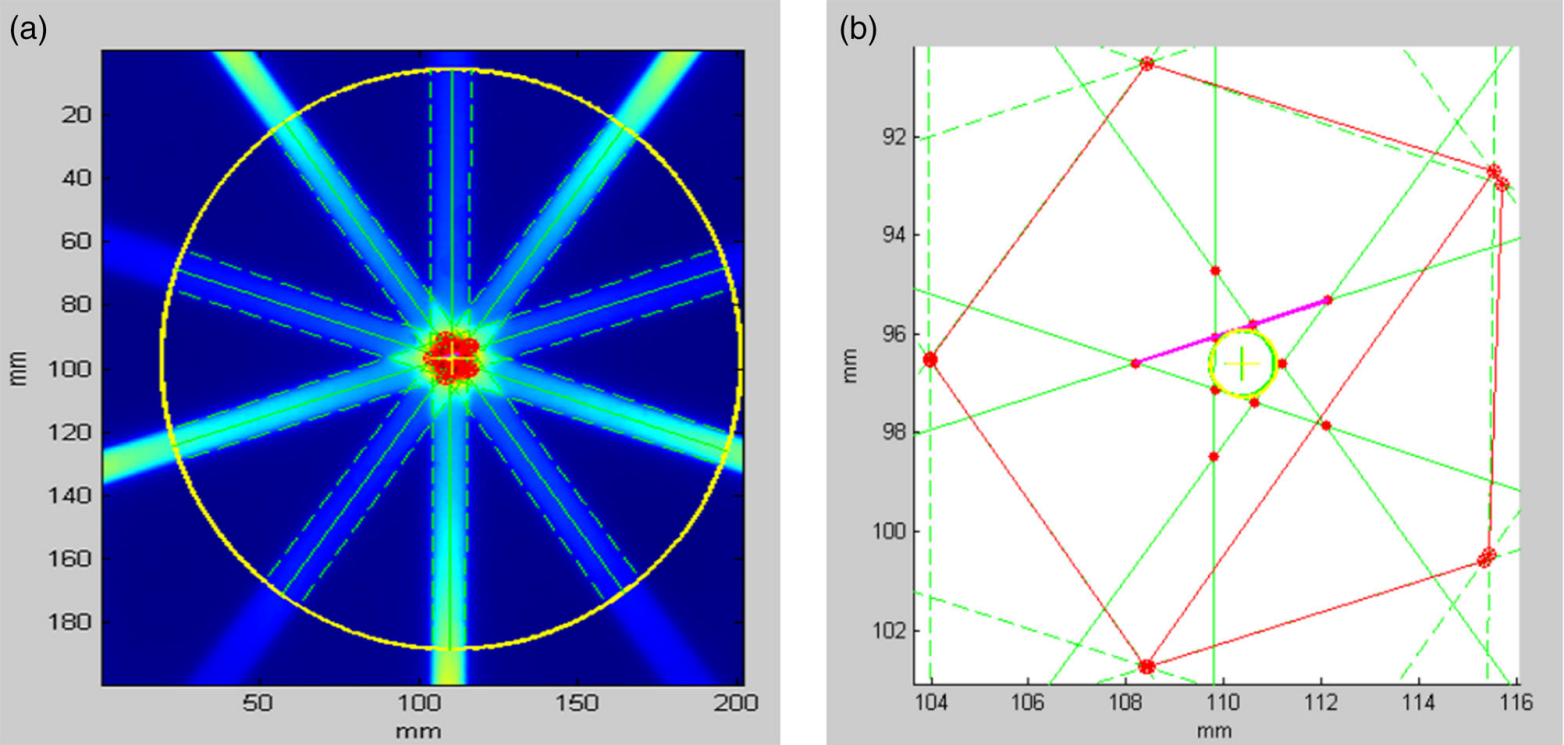
The film results for the gantry starshot test with the minimum tangent circle radius of 0.67 mm

Multiple MV images were taken with the field set to open only odd leaves and only even leaves. The positioning accuracy and repeatability were verified. The starshot test was performed to check the coincidence of radiation beams to a common isocenter under gantry rotation. Beam with the size of 1.25 × 2 cm^2^ was delivered at five different stationary gantry angles of 0°, 72°, 144°, 216°, 288° to the film sandwiched in between two 30 × 30 × 5 cm^3^ solid water blocks. The developed film result is shown in Figure 6, and the minimum tangent circle radius was 0.67 mm, which was within the suggested tolerance of 1 mm.

### 3.2 Beam characteristic and scan verification

#### 3.2.1 Percentage depth dose and relative beam profiles

**FIGURE 7:**
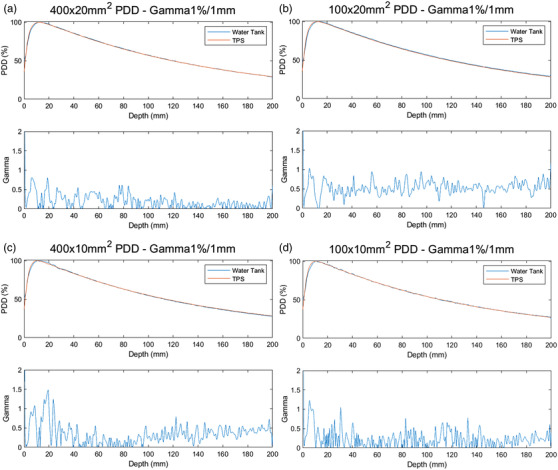
PDD curves of measurement and TPS calculation with 1D gamma analysis for: (a) 40 × 2 cm^2^ field; (b) 10 × 2 cm^2^ field; (c) 40 × 1 cm^2^ field; and (d) 10 × 1 cm^2^ field

Figure 7 shows PDD curves of measurement and TPS calculation with 1D gamma analysis for these fields. The measured PDD curves are overlapping the TPS calculated ones, showing an excellent agreement in terms of depth‐of‐dose maximum (*d*_max_) and dose fall‐off beyond *d*_max_. The measured and TPS modeled PDD_10_ were 55.7% and 54.8% for the 40 × 1 cm^2^ field, and 57.7% and 57.1% for the 40 × 2 cm^2^ field. The measured and TPS modeled PDD_10_ were 54.9% and 54.2% for the 10 × 1 cm^2^ field, and 57.0% and 57.0% for the 10 × 2 cm^2^ field. For all measured fields ranging from the size from 1.25 × 1 to 40 × 2 cm^2^, the mean PDD_10_ difference was −0.3% and the mean gamma (1%, 1 mm) pass rate beyond *d*_max_ depth was 94.9%.

**FIGURE 8:**
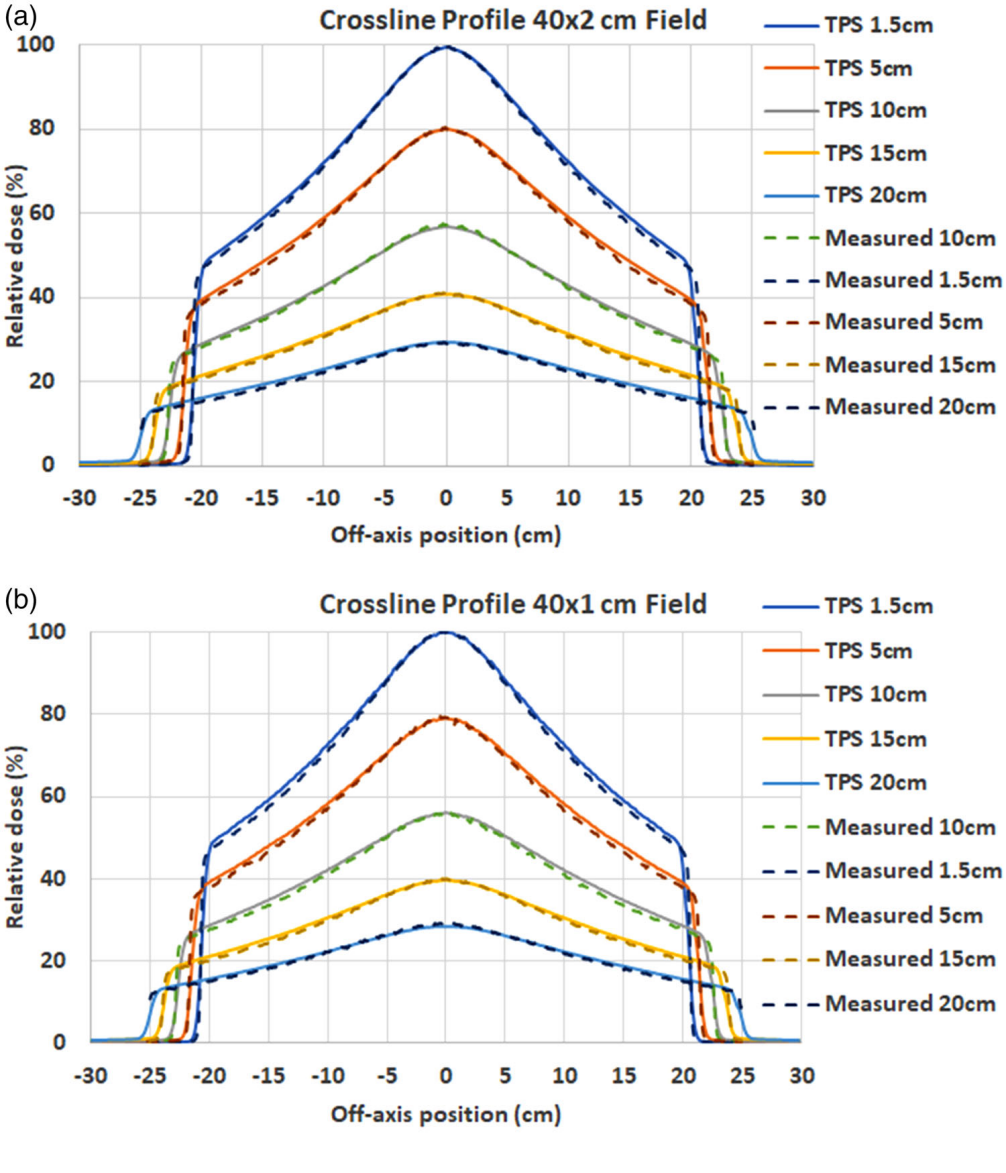
Measured and TPS calculated transverse (cross‐line) profiles of: (a) 40 × 2 cm^2^ field; and (b) 40 × 1 cm^2^ field at different depths

Selected transverse (cross‐line) profiles of measurement and TPS calculation for 40 × 2 cm^2^ and 40 × 1 cm^2^ fields at the depth of 1.5, 5, 10, 15, and 20 cm are shown in Figure 8. The measured and TPS modeled profile difference in the field core (80% of the nominal field) were 0.5% and 0.6% for the 40 × 2 cm^2^ field and 40 × 1 cm^2^ field, respectively, at all five depths. For all measured fields ranging from the size from 1.25 × 1 to 40 × 2 cm^2^, the mean profile differences in the field core were −0.3% ± 1.0% and −0.3% ± 1.2% for 2 cm and 1 cm jaw fields, respectively.

**FIGURE 9:**
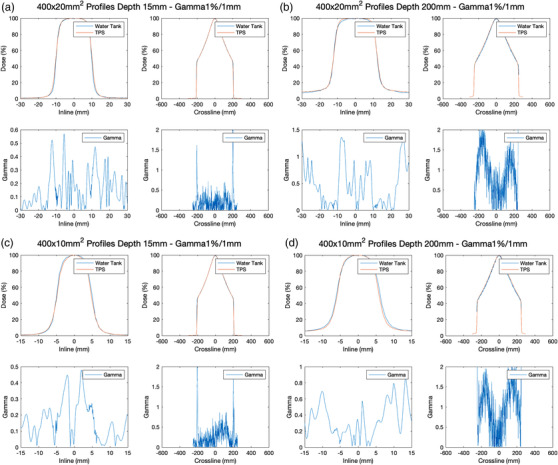
Measured and TPS calculated longitudinal (inline) profiles of: (a) 40 × 2 cm^2^ field at 1.5 cm depth; (b) 40 × 2 cm^2^ field at 20 cm depth; (c) 40 × 1 cm^2^ field at 1.5 cm depth; and (d) 40 × 1 cm^2^ field at 20 cm depth

The longitudinal (inline) profiles for fields ranging from the size from 1.25 × 1 to 40 × 2 cm^2^were measured and compared with the TPS calculation. Figure 9 shows the results of the 40 × 2 and 40 × 1 cm^2^ fields at the depth of 1.5 and 20 cm. The measured and TPS modeled differences in the FWHM of the longitudinal profiles were 0.2 and −0.4 mm for the 40 × 2 and 40 × 1 cm^2^ fields, respectively, at all five depths. The FWHM differences were −0.4 and −0.3 mm for the 40 × 2 and 40 × 1 cm^2^ fields, respectively, at all five depths. For all measured fields ranging from the size from 1.25 × 1 to 40 × 2 cm^2^, the mean and max FWHM differences were 0.3 and 0.4 mm for 2 cm jaw fields, and −0.3 and 0.5 mm for 1 cm jaw fields.

#### 3.2.2 Relative output factors

**FIGURE 10:**
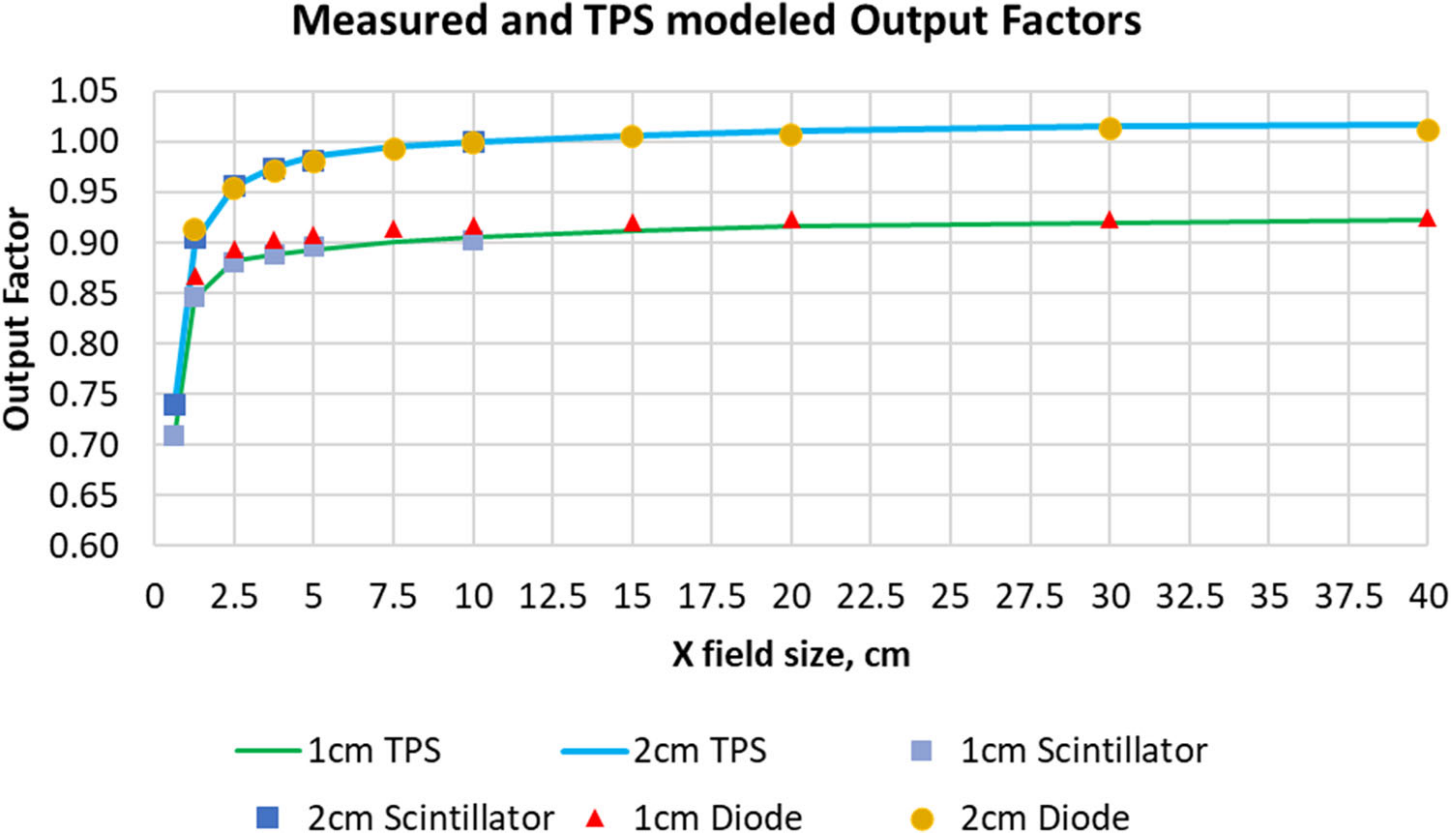
Diode, scintillator measured, and TPS modeled relative output factors in plastic water at 10 cm depth

The relative output factors were measured in plastic water at a depth of 10 cm using the Edge diode for larger fields in *x*‐direction from 1.25 to 40 cm, and W2 scintillator detector for smaller fields in *x*‐direction from 0.625 to 10 cm. Figure 10 illustrates the results for measured and TPS modeled output factors. As expected, the diode showed over responses relative to the scintillator for smaller fields up to 2%. The W2 measured output factors, serving as the ground truth for fields less than 10 cm in the *x*‐direction, increased from 0.706 to 0.903 as leaf opened from 0.625 to 10 cm at 1 cm y‐jaw opening and from 0.739 to 1 at 2 cm y‐jaw opening. The diode measured output factor, serving as the ground truth for fields greater than 10 cm in the *x*‐direction, increases from 0.918 to 0.925 as leaf opened from 10 to 40 cm at 1 cm y‐jaw opening and from 1.0 to 1.012 at 2 cm y‐jaw opening. The mean and max differences between TPS modeled and measured output factors for 2 cm jaw fields were −0.1% and 0.5%, respectively. The mean and max differences between TPS modeled and measured output factors for 1 cm jaw fields were 0.2% and 0.8%, respectively.

#### 3.2.3 Rotational output constancy and synchronicity

The Tomodose measured output constancy was within 0.213% for a stationary delivery at gantry angles of 0°, 90°, 180°, and 270°. The profile constancy of symmetry in transverse and longitudinal directions were within 0.2% and 0.44%, respectively, within the 2% suggested tolerance.

**FIGURE 11:**
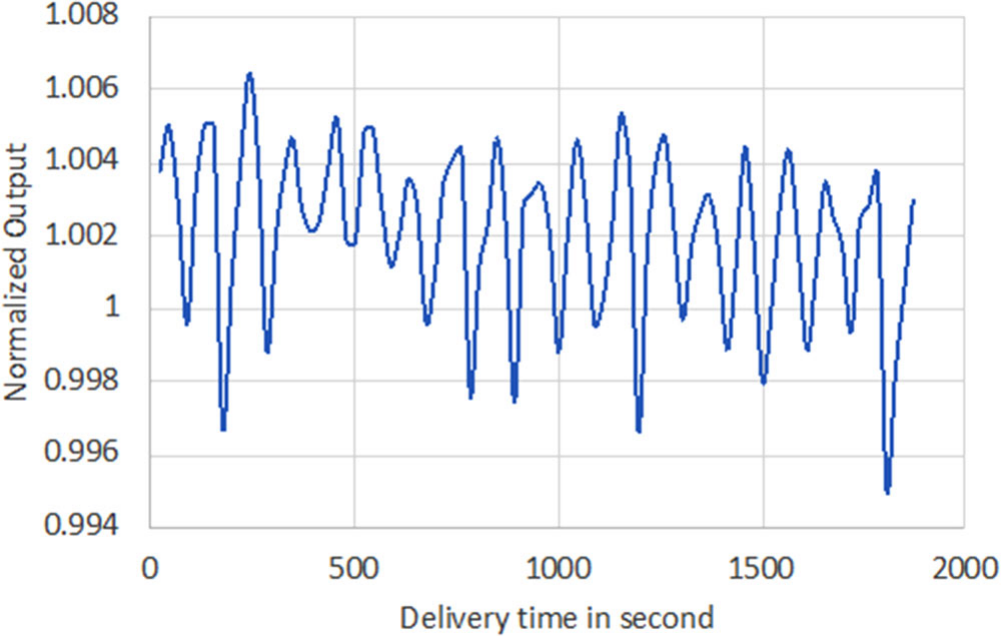
The recorded output versus delivery time for rotational output constancy test

Open beam with the gantry rotating continuously at 60 RPM was delivered for over 30 min to an ion chamber in a cylindrical phantom. The recorded output signal during the delivery is shown in Figure 11. The rotational output constancy was within 0.7% of the mean output during the 30 min delivery, with 2% suggested tolerance limit for the output variation with gantry angle.

**FIGURE 12:**
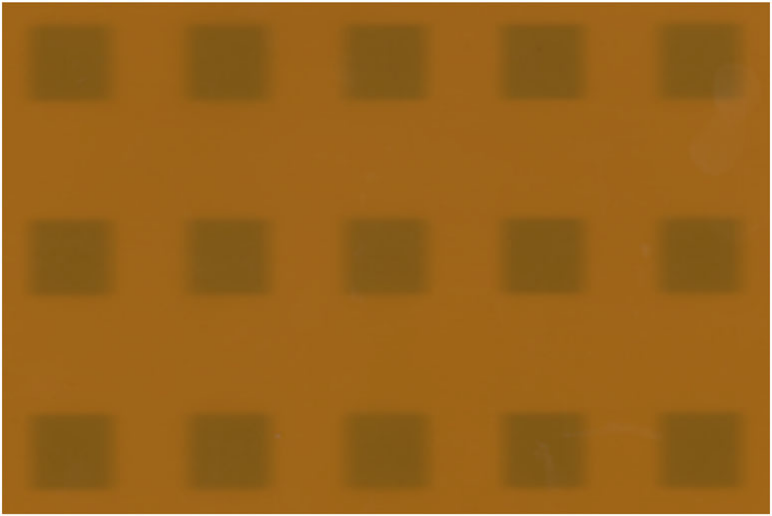
Film result of the synchronicity test with the max offset and angular deviation of 0.26 mm and 0.17°

The synchronicity plan was delivered to evaluate the accurate transmission of beams through the MLC to the isocenter in dynamic clinical delivery mode. Figure 12 shows the film result of the 15 rectangular exposures. The max offset and angular deviations are 0.26 mm and 0.17° compared to the expected values in the treatment plan within the tolerances of 0.5 mm and 0.5°.

#### 3.2.4 IMRT verification result

The AAPM TG‐119^12^ HN and prostate plans were measured using the ArcCHECK diode array to further evaluate the comprehensive dose delivery accuracy. Global gamma analysis of (3%, 2 mm) was performed with pass rates of 98.2% and 93.4% for the HN and prostate plans, respectively.

## 4 DISCUSSION

We have described our experience in the commissioning of the RefleXion X1 machine. The X1 presents some new challenges in commissioning, mostly related to its special small field geometry, high RPM gantry, and enclosed bore for mechanical checks. Based on TG‐148 suggestions, the mechanical and dosimetric performance of the RefleXion X1 machine has been commissioned and the accurate TPS model of the linac has been validated against the measurement data. Those data can serve as a baseline for routine quality assurance of the RefleXion X1 machine, as well as a reference for other institutions that are interested in introducing the RefleXion X1 machine into their IGRT and SBRT programs. Meanwhile, the fully validated TPS model can be a helpful tool for related BgRT studies in dosimetry, imaging, and quality assurance.

Even though the gantry rotation is 60 RPM, the X1 machine was very stable mechanically and was able to deliver beam accurately as verified by various tests presented in this study. All measured values were within the TG‐148 tolerances. Because of 1 and 2 cm jaw settings, extra care had to be taken for measuring the small field dosimetric parameters such as tighter centering and divergence thresholds of the field detector, minimizing the interference of the reference detector.

In addition to the tests presented, additional commissioning tests to validate the accuracy of the TPS modeling were performed according to the AAPM protocol TG‐53, and TG‐119 and TG‐244, including end‐to‐end tests using the ArcCheck (Sun Nuclear, Melbourne, FL, USA), motion interplay effects, inhomogeneity accuracy tests in lung and bone materials, and so forth. The commissioning of the kVCT, PET, and MV imaging systems were also carried out to ensure image quality and image guidance accuracy. Detailed results of these commissioning measurements are summarized in additional independent studies.

Future versions of the RefleXion X1 software will enable clinics to treat oligometastatic volumes with extended treatment field length in the superior–inferior direction. Additionally, as BgRT is not yet approved, there will be a need to include additional tests revolving around the biologically guided delivery components of the machine in the commissioning process, such as end‐to‐end tests.

## 5 CONCLUSION

This study represents the first commissioning and QA result of a clinical BgRT system, RefleXion X1 unit, and the validation of an accurate TPS model of the RefleXion linac. The data are especially useful for this newly developed machine that frequently uses a large amount of small segmented fields during the treatment delivery. A list of reference data are provided, which can be helpful for future RefleXion X1 machine commissioning in other institutions.

## AUTHOR CONTRIBUTIONS

Bin Han, Dante Capaldi, Nataliya Kovalchuk, Eric Simiele, John White, Daniel Zaks, Murat Surucu, and Lei Xing have all contributed to the measurement, data processing, and manuscript writing and editing.
